# Morphological and Motor Fitness Determinants of Shotokan Karate Performance

**DOI:** 10.3390/ijerph18094423

**Published:** 2021-04-21

**Authors:** Paweł Przybylski, Arkadiusz Janiak, Piotr Szewczyk, Dariusz Wieliński, Katarzyna Domaszewska

**Affiliations:** 1 Department of Various Sports and Camp Organisation, Poznan University of Physical Education, 61-871 Poznań, Poland; przybylski@awf.poznan.pl; 2 Faculty of Health Sciences, Calisia University, 62-800 Kalisz, Poland; a.janiak@akademiakaliska.edu.pl (A.J.); p.szewczyk@akademiakaliska.edu.pl (P.S.); 3 Department of Anthropology and Biometry, Poznan University of Physical Education, 61-871 Poznań, Poland; wielinski@awf.poznan.pl; 4 Department of Physiology and Biochemistry, Poznan University of Physical Education, 61-871 Poznań, Poland

**Keywords:** special motor fitness, speed, flexibility, endurance

## Abstract

The achievement of high performance levels in a complex structured sport such as karate is determined by the competitor’s physical fitness, fighting technique, tactics and mental state. This study aimed to identify the most important determinants of top-level performance in karate. Methods: The participants were 32 karate competitors (12 women and 20 men) aged 18–25 years. A series of tests measuring 11 anthropometric features was undertaken twice during a year, separated by a 6-week interval during a training camp at the Olympic Preparation Center in Walcz, Poland. Motor skills were measured with strength, speed, endurance, flexibility and reaction time tests. Special motor fitness was assessed with tests of karate technical skills. The results were subject to statistical analysis using multiple stepwise regression of the Polish Karate Federation ranking points as the dependent variable. Results: The multiple regression analysis revealed two main determinants of high scores in female and male karate competitors. In women, these were thigh circumference and the speed of the mawashi-geri-kick roundhouse technique (i.e., the maximum number of delivered kicks in 30 s), whilst for men they were the extent of the sideway leg swing to the highest possible height (yoko-geri) and general endurance assessed with the bent arm hang test. Conclusion: Karate training should account for the determinants of high-level competitive karate performance identified in this study. Strengthening the lower limbs, exercises increasing hip joint mobility, low position movements, performing leg techniques in various planes and applying external loads undoubtedly increase a karate athlete’s strength and lead to the development of a more extensive repertoire of karate leg techniques, especially at the highest-scoring head level (jodan).

## 1. Introduction

The martial art of karate requires highly developed technical skills, which, amongst others, include control of static and dynamic movements. Karate is a highly dynamic tournament sport. Both kumite and kata are karate techniques performed in an extremely dynamic, precise and coordinated fashion. Karate has a complex structure, and the competitors’ physical fitness, technique, tactics and mental state all affect successful performance [1,2]. During a kumite fight, attacks need to be executed at maximum speed to ensure that the attacker’s hand or the foot reaches its target without the opponent being able to respond [3]. Such fights are highly dynamic and intense [4]. Karate competitors’ actions are based on acyclic movements, and numerous muscle groups and all four limbs are engaged during combat. Karate fighters need to possess a great deal of strength to ensure that rapid movements are deployed with high precision. Attacking actions are often executed at very high speeds in karate training and competition, during which the fighters must strike before their opponents have any chance to defend themselves from an attack or counterattack. During combat, a karate contestant’s effectiveness is determined by multiple factors, such as on their adopted technique, tactics and motor skills [5,6]. Achieving championship-level performance requires undertaking long-term training to ensure the body is properly, morphologically and physiologically, conditioned. Successful performance in karate is known to depend on many factors, such as the competitor’s somatic build, fitness, level of technical preparation and mental state [4,7,8].

Becoming a sport champion is a long-term process that begins with the involvement of children and adolescents in sport. The first stage of a sports career is a functional component of competitive sport and it always lays the foundations for future championships. Maintaining an appropriate training system is key in championship building. Training should be optimized for achieving the best results and aimed at rationalizing the improvement of a contestant’s functions and skills at every stage of their biological and sporting development. Solutions are still being sought to develop the best selection criteria and to overcome limitations. Performance outcomes have always constituted such criteria, whilst limitations have been the principal determinants of the body’s biological development and functional capabilities. High-level sport performance can be only achieved by contestants in the right conditions. Selecting the appropriate morphological criteria for a given sport is vital because the body’s somatic structure is strongly determined by genetics, which can be modified by training only to a limited extent [9,10,11].

The long-term process of rationally targeted athlete training is determined by a model use of specific requirements for any given sport defined as target characteristics. Obviously, such requirements vary from sport to sport, including the rate at which a contestant is prepared, and are set against the functional capabilities of the developing body. From a holistic perspective, the optimal development of physical fitness is required for any athlete [12,13].

The correct assessment of the effects of movement stimulation depends on carrying out accurate and reliable diagnoses of an individual’s physical and psychomotor development at various stages of ontogenesis and training. Such diagnostic outcomes allow the development of optimal programming of various elements of sports training and the coordination of athlete’s motor skills, according to the goals set for each stage of sports advancement.

The methods of assessment of physical fitness have been developed for broadly understood karate and other combat sports. The most comprehensive approach is Specific Physical Fitness Tests (SPFT), consisting of the hip speed test, “jodan-tsuki” punch test, “chudan-tsuki”, “mawashi-geri” flexibility test, “mawashi-geri” kick test for the “jodan” level and flexibility test [14]. The SPFT was subsequently modified by Sterkowicz and Franchini [15], and Adamczyk et al. [14] proposed a modification of Story’s speed rating by assessing the strength measured with the bench press test in various body positions and endurance based on the number of circular kicks at the chest level in 90 s per punching bag. Koropanovski et al. [16] used the Sideward Leg Splits Test (SdLS) [17] for flexibility, acceleration and speed; the strength Countermovement Jump (CMJ), also used in judo; Standing Triple Jump (STJ); as well as the shuttle run for aerobic capacity assessment [18]. The same battery of tests was used by Koropanovski et al. [19] in their assessment of advanced athletes. The treadmill has also been used to assess aerobic capacity in trials following R.A. Bruce’s modified protocol. Anaerobic capacity has been assessed with the Wingate cycloergometer test [20]. Simple Reaction Time (SRT), Choice Reaction Time (CRT) and Decision Time (DT) ratings are also included in the test pool [21].

The aim of the present study was to determine the morphological and motor determinants of successful Shotokan karate performance in male and female practitioners.

## 2. Materials and Methods

The initial study pool consisted of 35 karate competitors; however, 3 were excluded due to injury. The number and classification of karate competitors are determined at the beginning of each year by the Board of the Polish Karate Federation. The participants were members of the World Karate Federation, who took part in major international competitions, e.g., the World Championships, European Championships and Olympic Games.

The study group comprised 32 karate competitors (12 women and 20 men) aged 18–25 years ranked from 1 kyu to 3 Dan. A series of tests was performed twice (i.e., at two stages) during one year, at an interval of 6 weeks during training camp events at the Olympic Preparation Center in Walcz, Poland. Eleven anthropometric features were measured describing the basic characteristics of the contestants’ somatic build. Physical fitness was measured with the use of 16 motor skills tests of strength, speed, endurance, flexibility and reaction time. Special motor fitness was assessed with 5 tests of karate-specific technical skills, measuring special speed, endurance, and flexibility. The study was conducted in accordance with the Declaration of Helsinki and the National Statement of Intent as well as the Human Research Ethics Guidelines, and was approved by the Institute for Research in Biomedicine of the Poznan University of Medical Sciences (2008-11-13; Ethics Approval Number: 1101/08). All participants provided their written consent to take part in the tests after familiarizing themselves with the study protocol.

The study was conducted during the first 3 days of the athletes’ stay at the Olympic Preparation Centre, in the same sequence, on each study day. On the first day, anthropometric characteristics of athletes’ somatic build were measured, including the times of simple and complex reaction to optical and acoustic stimuli. On the second day, the remaining tests of physical fitness were carried out, with the exception of the athletes’ endurance assessment. On the third day, karate-specific tests of special motor fitness, special speed, special endurance and special flexibility were performed. The Beep-Test was carried out as the last measurement. On each day, the tests were performed 2 h after a standard breakfast.

### 2.1. Anthropometrics

Body mass and body height were measured with the WPT 60/150 OW medical scales (Radwag^®^, Radom, Poland) to the nearest 100 g. The first author of the study carried the anthropometric measurements of the circumferences of the arm, forearm, thigh and lower leg as well as the length of the upper and lower limbs using a tape measure graduated in 0.1 cm increments, following the procedure in Norton [22]. The thickness of three skinfolds was also measured at the right side of the body using a Harpenden Skinfold Caliper to the nearest 0.1 mm (graduation 0.2 mm, range 80 mm) at the following sites: suprailiac (immediately superior to the iliac crest in midaxillary line), subscapular (the undermost tip of the inferior angle of the scapula) and the calf (a vertical pinch parallel to the long axis of the leg). The observed differences between the three measurements of body weight, body height, and limb length were <1%. The differences in skinfold measurements amounted to <5%.

### 2.2. Physical Fitness

The karate athletes’ physical fitness was assessed with tests measuring the main motor skills, such as strength, speed, endurance, flexibility and reaction time. There were nine assessments of strength (static, dynamic, functional and maximal) as well as a hand movement speed test, flexibility test (torso forward), endurance test (Beep-Test) and criterion-related 20-m shuttle run test for estimating cardio-respiratory fitness [23]. There were also five tests measuring the time of simple and complex reactions of the limbs (responses to optical and acoustic stimuli). Sixteen tests assessing all round fitness were carried out in total. An in-house time counter with an electronic auxiliary MC-2 device (VOLTCRAFT^®^, Lindenweg, Hirschau/Germany) as well as an electronic device with an auxiliary MCZR/ATB 1.0 light (INFO-ELEKTRO, Ruda Śląska, Poland) set were used. Both devices were designed at the Research and Teaching Aid Department of the University of Physical Education in Poznan. For tests involving more than 1 repetition, each successive trial was performed in resting conditions, i.e., after the heart rate returned to baseline, so that each trial was performed under the same conditions.

#### 2.2.1. Simple Reaction Time of the Feet (Optical Stimulation)

Each participant had to react as quickly as possible to 10 consecutive light impulses by pressing a foot against the pedal attached to a special platform on the ground. The arithmetic mean of the times was used for analysis after cutting off the three top and the three bottom times [21].

#### 2.2.2. Simple Reaction Time of the Hands (Optical Stimulation)

The measurements were performed to the nearest 0.001 s. Each participant had to react as quickly as possible to 10 consecutive light impulses by pressing a button on a special hand-held handle with a thumb. The arithmetic mean of the times was used for analysis after cutting off the three top and three bottom times [21].

#### 2.2.3. Complex Reaction Time of the Upper Limbs (Optical Stimulation)

The measurements were performed with an accuracy of 0.01 s. Each participant had to react as quickly as possible to 15 consecutive light impulses by pressing the buttons on hand-held handles with the thumbs. Each hand corresponded to a respective color. If an orange bulb was lit, no reaction was to be produced. The arithmetic mean of the times was used for analysis after cutting off the three top and the three bottom times [21].

#### 2.2.4. Complex Reaction Time of the Upper and Lower Limbs (Visual and Acoustic Stimulation)

The measurements were performed with an accuracy of 0.01 s. Each participant had to react as quickly as possible to 10 consecutive light and sound impulses, pressing the thumbs on the buttons on hand-held handles, and the feet on the pedals mounted on a special platform on the ground. Each limb was assigned only one type of pulse. The arithmetic mean of the times was used for analysis after cutting off the three top and three bottom times [21].

#### 2.2.5. Hand Movement Speed (Disc Tapping)

Each participant was to touch alternately two spaced disks with the more efficient hand as fast as possible. A participant, standing in front of the table, put his or her less efficient hand on a rectangle in the middle between the disks. At the command “Start” he or she made 25 movements back and forth (a total of 50 touches). Every touch was loudly counted, and the command “Stop” ended the test. The test was performed twice and the better score was recorded. The results were measured to the nearest 0.1 s.

#### 2.2.6. Strength Measurement

Participants performed a forward throw with a swing of a 2 kg medicine ball while standing in front of a line. The ball was held behind the head with both hands. If, after the throw, the participant crossed the throwing line, the test was not scored. Each participant was allowed three attempts and the measurements were taken with an accuracy of 0.1 m. The better result was used for analysis.

#### 2.2.7. Grip Strength

Hand grip strength was measured using a manual dynamometer (Lafayette 78010, Lafayette Instrument Company, Lafayette, IN, USA). Each participant took an upright posture with the arms at the sides, not touching the body, slightly bent at the elbows during the measurement [24]. The test was repeated three times with a 1-min break between the attempts to avoid the effects of muscle fatigue. Both the right and the left hands were tested, and the highest score by the hand declared as dominant was used for further analysis. The accuracy of the measurement was 0.01 kg.

#### 2.2.8. Long Jump

Participants performed a standing broad jump on a mattress. Standing with their legs astride in front of the starting line each participants bent their knees and swung their arms backwards. After take-off, each participant attempted to make the longest possible jump possible and land on both feet. The test was performed 3 times and the best result was used for analysis.

#### 2.2.9. Bent Arm Hang

With the examiner’s assistance, each participant performed a pull up on a 2.5 mm diameter bar, with the arms bent in such a way that the chin was at the level of the bar. In this position, each participant tried to hold on the bar as long as possible. The test was completed when the participant’s eyes were at the same level as the bar. The hang time was measured to the nearest 0.1 s.

#### 2.2.10. One Repetition Maximum Bench Press Test (1 RM Test (One Repetition Maximum Bench Press Test))

The athletes lay down on a horizontal bench with their legs bent at a right angle and the feet resting on the ground. Each participant gripped the barbell placed at a distance of shoulder width using both hands. Next he or she lifted the barbell from the rack, lowered it on the chest, and lifted it up with the upper limbs fully extended. After the test the participant put the barbell back on the rack, with assistance if necessary. The starting weight was determined individually by each athlete, and the progression of the barbell load was 2.5 kg. Two failed attempts excluded the athlete from the test.

#### 2.2.11. Attainable flexure

With the hands clasped together at the nape of the neck, in a supine position on the mattress, the participant touched the mat with their back and then returned to the supine position, with their elbows touching the knees. The feet rested on the mat and the test consisted of a maximum number of repetitions in 30 s.

#### 2.2.12. High Jump

Each participant stood on a box equipped with a centimeter scale on one of its walls, embracing the box edge with the toes. The standing surface level was marked as 0. With the legs straightened in the knee joints and arms straightened down, the participants performed a maximum downward bend. The score was marked on the front wall of the box when the lowest position was reached. If the fingers of both hands were not on the same line, the mean distance for both hands was considered for analysis. The test was performed 3 times and the best result was used for analysis.

#### 2.2.13. Endurance Testing: Beep Test

The test took place in a sports hall. The participants completed a number of 20 m runs from one line to another. The test started on a sound signal, and the running pace was determined by time intervals defined by successive sound signals. After each beep, the runner had to cover a distance of 20 m. The starting pace was very slow but kept increasing with the changing attempt levels. The runner had to maintain the pace, and the test ended when the participants exceeded the time limit necessary to complete the given distance twice [23].

### 2.3. Special Motor Fitness

The test aimed to assess the participants’ karate-specific skills. Each participant delivered punches that were counted by the instructor and recorded with a video camera placed on a fixed tripod at the participant’s side at a right angle. Special speed was measured with the choku-tsuki test (maximum number of straight hand punches performed in 10 s); special endurance test with the maximum number of uraken-uchi + gyaku-zuki combined attacks executed in 30 s and the mawashi-geri test (maximum series of kicks performed in 30 s); and special flexibility with the yoko-geri test (a side kick up to a maximum height, so-called ‘Turkish twine’). In total, 5 tests were used to assess the karate competitors’ special motor fitness [25,26,27].

### 2.4. Statistical Analysis

Summary statistics consisted of means and standard deviations (with 95% confidence limits). The Shapiro–Wilk test was used to test for normality of the distribution. Data were subjected to multiple stepwise regression analysis in which the best set of independent variables was selected and the coefficients of determination (R squared) were estimated for the variables in the model. Lambda ridge regression was also performed, which is a more stringent test that takes into account the variance around the coordinates’ origin. It often allows those model determinants present in excess to be eliminated as well as any variables of minimal significance. All in all, the set of independent variables thus contained 33 items. The dependent variable was the number of ranking points awarded to each karate competitor by the Polish Karate Federation (PKF) according to official regulations. The PKF introduced a ranking points system in 2006. The points are used to determine the best athletes in particular competitions and weight categories and are the basis for appointing competitors to the national karate team. All karate athletes participating in the tests took part in the same competitions. It should be noted that the Poland national team members are obliged to participate in competitions in which ranking points are awarded, except for cases when a competitor is injured.

The degrees of correlation were set according to the following ranges: <0.10 trivial, 0.10 to 0.30 small, 0.30 to 0.50 moderate, 0.50 to 0.70 large, 0.70 to 0.90 very large and 0.90 to 1.00 almost perfect [28]. The level of statistical significance was set at *p* < 0.05 in all cases. The obtained results were analyzed statistically using the Dell Statistica data analysis software system (version 13, software.dell.com, Dell Inc., Round Rock, TX, USA).

## 3. Results

Table 1 presents the anthropometric and physical fitness characteristics and special motor fitness test results of the karate competitors under study.

The independent variables comprised the determinants of sport performance that generally described the contestants’ morphological features, physical fitness and special motor fitness.

Most of the variables considered ‘independent’ were, in fact, logically speaking, dependent on many factors; however, this is beyond the scope of this paper. The multi-stepwise regression allowed the best set of independent variables to be chosen and to precisely establish the sets of factors responsible for sporting outcomes in karate as well as their relative importance as determinants. In addition, a similarly acting lambda ridge regression was performed, which is, however, a more stringent procedure, which explained the variance around the coordinates’ origin. It also allowed for rejecting those determinants that excessively or insignificantly contributed to the general model.

Six features were found to fit the model in the first part of the study for female participants, using stepwise multiple regression that, jointly in effect, determined the dependent variable at 100%. Each subsequent variable added was decreasingly less and less significant, but when taken together they practically accounted for the dependent variable in its entirety. Thigh circumference was found to be the most significant variable at 76% (R-squared), followed by the foot kicking rate (mawashi-geri) at 19% (R-squared change); the remaining independent variables taken together accounted for only 4% (Table 2).

Likewise, the model also included six features in the second part of the study that when taken together practically determined the dependent variable in its entirety (100%). It was found that three features fit the model in both the first and second stages of the study (i.e., thigh circumference, mawashi-geri and long jump), while the other three did not (Table 3 and Table 4).

As expected, the ridge regression left two determining variables in the model. The multiple regression analysis and the consistency of outcomes from the first and second parts of the study on female karate competitors revealed two basic variables with a decisive influence on high karate performance: thigh circumference and mawashi-geri (i.e., the maximum number of kicks performed in 30 s) (Table 5).

Table 6 presents determinants of karate performance as defined by the number of ranking points. The model includes 10 characteristics that together determine the dependent variable as a whole (100%). Table 7 lists the determinants of karate performance in the order in which the variables enter the multiple regression model in consecutive “steps”. The most significant determinant was yoko-geri, which explained the dependent variable in 64% (R-square), followed by lower limb length at 13% (R-square change) and lower leg circumference at 20%. The remaining independent variables collectively accounted for about 2% of the determining factors; thus, like in the case of female karate competitors, they can be ignored. Table 8 presents the results of the multiple stepwise regression analysis of the second series test results determining karate performance. The model includes 9 characteristics determining the dependent variable in almost 100%. Three variables in the model were the same as for the first series tests (yoko-geri, bent arm hang and arm circumference).

The multiple regression analysis results and the consistency of the results of the first and second series of tests revealed two variables with a decisive impact on the sport performance of male karate competitors: yoko-geri (side kicks up to the highest possible height) and bent arm hang determining overall endurance (Table 9). It can be concluded that male karate competitors with significantly high endurance levels, i.e., ability to fight without fatigue and accurately hit the opponent in upper body areas (head and neck), will have a greater chance of achieving top results in karate.

## 4. Discussion

The aim of the study was to identify and assess the morphological and motor fitness determinants of successful performance in karate. Most combat sports require a combination of technique, strength, aerobic fitness, power, and speed. In general, no single trait dominates in combat sports.

The multiple regression analysis revealed that two variables had the greatest impact on the karate performance of male and female competitors. For women, these were thigh circumference and the speed of the mawashi-geri-kick roundhouse technique (i.e., the maximum number of delivered kicks in 30 s), whilst for men they were the extent of the sideway swing to the highest possible height (yoko-geri) and overall endurance assessed with the bent arm hang test.

Because the actions of the upper limbs are more precise and faster than the actions of the lower limbs during karate tournament fights, the advantages of the former are clear, as observed by Chaabene et al. [5]. Nevertheless, the high-level-jodan foot techniques were scored the highest as having the greatest assigned levels of difficulty [29,30]. Chaabene et al. also pointed out that multivariate studies are needed in modern sport to assess the impact of various factors on karate performance; they are also extremely important in furthering the knowledge of the interrelationships between these methods [5].

The results of studies assessing simple or choice reaction times between successful and less successful karate athletes are also inconclusive. Fontani et al. [31] found that athletes with 3rd and 4th Dan black belts had shorter reaction times to a stimulus than athletes with 1st and 2nd Dan black belts. In contrast, Mori et al. [32] in their study four years earlier showed no significant difference in simple reaction time between athletes of varying levels of proficiency. In the present study, reaction time was not a significant determinant of high-level performance. Modern karate performance requires high levels of physical fitness, particularly flexibility, which involve a high degree of joint movement and ensures that the fighting technique is properly executed.

There are different determinants of ranking positions in women and men practicing karate [33]; however, the literature on karate performance determinants in female athletes is rather limited. Women’s success in karate is strongly determined by morphological traits (i.e., strong thighs) together with the rapidity and strength of the kicks; whereas for men, it is endurance in combat and execution of accurate hits on the opponent’s body in high-scoring areas. Our results demonstrate that sports training is indeed mainly focused on those important variables. Combining variables allows a broader consideration of determinants in Shotokan karate. Undoubtedly, the most significant determinant factor of successful karate performance for both sexes appears to be ideally developed strength based on the morphology of the limbs. These elements are strongly related to each other and are commonly termed the as ‘the arm and leg strength factor’. This is essentially the one and only determinant of karate success in women, whilst for men it is one of the main determinants, followed by the range of mobility in the joints of the upper and lower limbs when correlated with the complex responses of the limbs. A study by Katić on karate learners showed that successful karate performance was mainly achieved thanks to their level of knowledge and ability to efficiently execute karate technique(s) as conditioned by general and specific motor skills [34,35]. The present study also identified a group of morphological, positive and negative, determinants of effective karate performance in senior contestants in reference to the used karate techniques [36]. Other authors examined the effect of cardiorespiratory performance and maximal power output (Wingate test) on the performance level. Both factors did not significantly differentiate between karate athletes at the international and national levels, despite the fact that VO_2max_ plays an important role in preventing fatigue during training and accelerates recovery between combats.

Many studies have confirmed that high-level karate performance is generally related to the explosive strength of the muscles. Furthermore, highly competitive karate athletes require both rapid acceleration and deceleration of body segments and muscular explosive power training [37,38,39]. Koropanovski et al. [16] in their study of morpho-functional differences between kata and kumite athletes also showed that a higher explosive power could be beneficial for kumite, while both smaller stature and higher flexibility (particularly of the lower extremity) could be important for the exceptionally low postures of the kata competitors.

Highly developed strength determines the successful performance in Shotokan karate based on the morphological properties of the limbs. Indeed, in women, this constitutes the basic and only determinant of success in karate, whereas in men it is one of a number of leading factors, closely followed by the range of mobility in the joints of the upper and lower extremities. Women’s success in karate depends largely on their thigh strength as well as the speed and strength of strikes/blows inflicted with the lower limbs, albeit in men, successful karate performance is largely determined by their endurance in combat and accurate hits to the opponent’s high-scoring body areas. Karate training should thus focus mainly on these factors to enable novice athletes to achieve success in competition. Undoubtedly, strength can be increased by the strengthening of the lower limbs, hip joint mobility exercises, low position movements, execution of leg techniques in various planes, and the use of external loads. All this will thereby permit a wider repertoire of leg techniques to be acquired, especially at the highest-scoring jodan-head level.

It can be concluded that these dimorphic differences in determinants of successful performance in Shotokan karate may have their evolutionary foundations. Strength is an evolutionary motor trait assigned to the male sex, and flexibility (or greater mobility in the joints) to the female sex. Karate as a sport paradoxically combines both these elements. Thus, each sex during karate training refines these evolutionarily unassigned properties, and their development guarantees an advantage in one’s gender category and thus competitive success. A strong woman is likely to win over a weaker woman while both are naturally flexible; a more “flexible” man will win against a “stiffer” man, while both are naturally equally strong.

Limitation of the study: The limitation of this study was the unequal number of female and male participants. It was also impossible to carry out physiological tests such as VO_2max_ or Wingate test to assess participants’ anaerobic capacity. Blood indicators of the body’s response to the applied training loads were not analyzed either.

## 5. Conclusions

Systematic analysis of the athlete’s motor level during training affects the individualization and optimization of the training process. Multiple regression analyses have shown that, in the case of both men and women, two significant variables determine high-level Shotokan karate performance to the greatest extent. In the studied group of women, they were the thigh circumference and the speed of the mawashi-geri. In men, they were the yoko-geri range and overall endurance measured with the bent arm hang test. In the case of women, successful karate performance has a strong morphological basis (strong thighs) and depends on the speed and strength of the kicks. In the studied group of men, it was endurance in combat and the precision of hitting the opponent in the highest-scoring body areas. The study results indicate that it is beneficial to focus on the above determinants in the process of training of karate athletes. These results can be directly used in the selection of athletes with appropriate motor skills, predispositions, or anthropometric features. This will allow the proper qualification of young athletes to the respective training groups and guide their sport development towards the attainment of the championship level.

The research is cross-sectional. We realize that not all variables can be controlled in our study. It seems logical to think that people with more practice time had more opportunities to be exposed to the competitive environment. In fact, practice is directly related to performance and learning. Williams and Elliot [40] showed that expert martial artists have greater anticipation skills, suggesting that perceptual skill in karate depends on task-specific knowledge structures acquired through experience.

## Figures and Tables

**Table 1:** Mean values of the anthropometric and physical fitness characteristics and special motor fitness test results of male and female karate competitors.

Variable	n (20) Men		n (12) Women	
	M ± SD	95% CI	M ± SD	95% CI
Age (years)	20.40 ± 4.16	(18.45–22.35)	20.92 ± 3.00	(19.01–22.82)
Height (cm)	176.25 ± 6.31	(173.30–179.20)	165.08 ± 6.11	(161.20–168.97)
Weight (kg)	70.35 ± 8.14	(66.54–74.16)	58.58 ± 5.52	(55.08–62.09)
Lower limb length (cm)	91.75 ± 4.14	(89.81–93.69)	87.00 ± 3.28	(84.92–89.08)
Upper limb length (cm)	77.30 ± 3.73	(75.55–79.05)	73.08 ± 3.23	(71.03–75.14)
Thigh circumference (cm)	53.85 ± 4.33	(51.82–55.88)	55.58 ± 3.42	(53.41–57.76)
Shin circumference (cm)	38.15 ± 2.68	(36.90–39.40)	36.33 ± 1.92	(35.11–37.55)
Arm circumference (cm)	32.15 ± 4.06	(30.25–34.05)	28.33 ± 2.39	(26.83–29.85)
Forearm circumference (cm)	28.40 ± 2.90	(27.38–29.42)	25.00 ± 1.60	(23.99–26.01)
Shoulder skinfold (mm)	11.36 ± 2.64	(10.12–12.59)	11.52 ± 1.63	(10.48–12.55)
Stomach skinfold (mm)	10.07 ± 1.72	(9.27–10.87)	10.52 ± 2.04	(9.22–11.82)
Shin skinfold (mm)	8.54 ± 1.31	(7.93–9.15)	8.83 ± 1.04	(8.17–9.50)
Simple reaction time: right leg (optical stimulation) (s)	0.28 ± 0.04	(0.26–0.29)	0.29 ± 0.03	(0.27–0.31)
Simple reaction time: left leg (optical stimulation) (s)	0.27 ± 0.02	(0.25–0.28)	0.28 ± 0.03	(0.26–0.30)
Simple reaction time: hands (optical stimulation) (s)	0.23 ± 0.01	(0.22–0.23)	0.23 ± 0.02	(0.22–0.24)
Simple reaction time: hands (acoustic stimulation) (s)	0.15 ± 0.02	(0.14–0.16)	0.16 ± 0.02	(0.14–0.17)
Complex reaction time: hands (s)	0.31 ± 0.04	(0.29–0.33)	0.30 ± 0.03	(0.28–0.32)
Complex reaction time: hands + legs (s)	0.39 ± 0.05	(0.37–0.42)	0.41 ± 0.09	(0.35–0.47)
Disk tapping (number)	93.30 ± 6.22	(90.39–96.21)	95.00 ± 11.78	(87.52–102.48)
Medicine ball throw (cm)	1147.00 ± 201.50	(1052.70–1241.30)	845.00 ± 115.17	(771.83–918.17)
Grip strength (kg)	44.30 ± 9.55	(39.83–48.77)	29.00 ± 3.62	(26.70–31.30)
Long jump (cm)	254.85 ± 20.18	(245.40–264.30)	215.08 ± 15.01	(205.55–224.62)
Bent arm hang (s)	46.60 ± 9.35	(42.22–50.98)	30.42 ± 14.43	(21.25–39.58)
Bench press 1 RM test (kg)	65.50 ± 15.89	(58.07–72.93)	40.00 ±10.66	(33.23–46.77)
Flexure to sit down (amount)	41.70 ± 4.86	(39.43–43.97)	37.83 ± 6.04	(33.99–41.67)
Attainable flexure (cm)	21.20 ± 4.80	(18.95–23.45)	17.08 ± 6.47	(12.97–21.20)
High jump (cm)	55.75 ± 8.83	(51.62–59.88)	42.17 ± 6.39	(38.10–46.23)
Beep Test	57.28 ± 6.25	(54.36–60.20)	49.90 ± 9.53	(43.85–55.95)
Straight punch-choku-zuki (number of punches)	77.10 ± 10.15	(72.35–81.85)	71.00 ± 12.55	(63.03–78.97)
Hand punching combinations: uraken-uchi + gyaku-zuki, i.e., reverse fist +reverse punch (number of punches)	74.60 ± 8.95	(70.41–78.79)	64.00 ± 9.15	(58.19–69.81)
Mawashi-geri, i.e., spin kick (number of kicks)	55.90 ± 5.26	(53.44–58.36)	46.25 ± 7.14	(41.72–50.78)
Leg upswing (side kick) (cm)	175.52 ± 11.50	(170.52–181.28)	159.17 ± 12.45	(151.25–167.09)
Cross-sectional stride (cm)	22.60 ± 11.45	(17.24–24.00)	13.92 ± 7.59	(9.10–18.74)

Results expressed as M = mean, SD = standard deviation (confidence intervals 95%).

**Table 2:** Summary regression details from the 1st study stage for female participants; dependent variable = ranking points.

Determinant/Feature	Ensuing Stage	Multiple Correlation	Cumulative Coefficient of Determination	Partial Coefficient of Determination	*p*
		R	R- Squared	R- Squared	
Thigh circumference (cm)	1	0.8746	0.7649	0.7649	0.1416
Mawashi-geri, i.e., spin kicks (maximum number)	2	0.9802	0.9609	0.1960	0.1254
Choku-zuki, i.e., straight punches (maximum number)	3	0.9907	0.9815	0.0206	0.2811
Long jump (cm)	4	0.9981	0.9962	0.0147	0.1800
Flexure to sit down (number)	5	0.9998	0.9996	0.0033	0.1377
Bench press 1 RM test (kg)	6	1	1	0.0003	0.0189

**Table 3:** Summary regression details from the 2nd study stage for female participants; dependent variable = ranking points.

Determinant/Feature	Ensuing Stage	Multiple Correlation	Cumulative Correlation	Partial Correlation	
		R	R- Squared	R- Squared	*p*
Thigh circumference (cm)	1	0.8705	0.7579	0.7579	0.1443
Mawashi-geri, i.e., spin kicks (maximum number)	2	0.9713	0.9435	0.1856	0.1538
Long jump (cm)	3	0.9853	0.9709	0.0273	0.3025
Leg upswing (cm)	4	0.9950	0.9901	0.0192	0.2489
Shoulder skinfold (mm)	5	0.9990	0.9980	0.0078	0.2133
High jump (cm)	6	1	1	0.0019	0.0073

**Table 4:** Summary details of lambda ridge regression from the 1st study stage for female participants; dependent variable = ranking points.

L = 0.10000, R = 0.94747702, R^2^ = 0.89771271					
F(2.5) = 21.941 *p* < 0.00335, Estimated Standard Error: 106.04					
	Standardized Regression Coefficient	Standard Error	Unstandardized Regression Coefficient	Standard Error	
	BETA	BETA	B	B	*p*
Free W	1760.59			843.884	0.0913
Thigh circumference (cm)	−0.6100	0.1485	−47.7082	11.615	0.0092
Mawashi-geri, i.e., spin kicks (number of kicks)	0.4670	0.1485	23.4520	7.4577	0.0255

**Table 5:** Summary details of lambda ridge regression from the 2nd study stage for female participants; dependent variable = ranking points.

L = 0.10000, R = 0.96733337, R^2^ = 0.93573385					
F(4.3) = 10.920 *p* < 0.03916, Estimated Standard Error: 108.51					
	Standardized Regression Coefficient	Standard Error	Unstandardized Regression Coefficient	Standard Error	
	BETA	BETA	B	B	*p*
Free W.			1984.509	914.2573	0.1183
Thigh circumference (cm)	−0.5172	0.1888	−41.352	15.0940	0.0713
Mawashi-geri, i.e., spin kicks (maximum number)	0.3119	0.1754	13.2209	7.4355	0.1734
Hand punching combinations; uraken-uchi + gyaku-zuki, i.e., reverse fist + reverse punch (number of punches)	0.3215	0.2011	9.7345	6.0887	0.2081
Forearm circumference (cm)	−0.2041	0.1692	−32.274	26.7466	0.3140

**Table 6:** Summary regression details from the 1st study stage for male participants; dependent variable = ranking points.

Determinant/Feature	Ensuing Stage	Multiple Correlation	Cumulative Coefficient of Determination	Partial Coefficient of Determination	
		R	R- Squared	R- Squared	*p*
Leg upswing (cm)	1	0.7997	0.6395	0.6395	0.1483
Lower limb length (cm)	2	0.8787	0.7721	0.1326	0.2622
Shin circumference (cm)	3	0.9874	0.9750	0.2028	0.0785
Flexure to sit down (number)	4	0.9931	0.9864	0.0113	0.2492
Bench press 1 RM test (kg)	5	0.9966	0.9932	0.0068	0.2462
Shinfold thickness (mm)	6	0.9979	0.9959	0.0027	0.3169
Complex reaction time: hands (s)	7	0.9994	0.9989	0.0029	0.1863
Forward flexure (cm)	8	0.9999	0.9998	0.0009	0.1348
Bent arm hang (s)	9	0.9999	0.9999	0.0001	0.1009
Arm circumference (cm)	10	1	1	6.38 × 10^-6^	0.0058

**Table 7:** Summary regression details from the 2nd study stage for male participants; dependent variable = ranking points.

Determinant/Feature	Ensuing stage	Multiple Correlation	Cumulative Coefficient of Determination	Partial Coefficient of Determination	
		R	R- Squared	R- Squared	*p*
Leg upswing (cm)	1	0.7387	0.5457	0.5457	0.1879
Complex reaction time hands + legs (s)	2	0.8680	0.7534	0.2077	0.2340
Bent arm hang (s)	3	0.9276	0.8605	0.1071	0.2591
Arm circumference (cm)	4	0.9609	0.9233	0.0628	0.2696
Forearm circumference (cm)	5	0.9873	0.9748	0.0514	0.1927
Simple reaction time (optical stimulation): right leg (s)	6	0.9978	0.9956	0.0207	0.1437
Straight punch-choku-zuki (number)	7	0.9999	0.9999	0.004	0.0556
Cross-sectional stride (cm)	8	0.9999	0.9999	9.19 × 10^-5^	0.1207
Forward flexure (cm)	9	1	1	6.67× 10^-6^	0.0795

**Table 8:** Summary details of lambda ridge regression from the 1st study stage for male participants; dependent variable = ranking points.

L = 0.10000, R = 0.92555474, R^2^ = 0.85665159					
F(3.8) = 15.936 *p* < 0.00098, Estimated Standard Error: 29.385					
	Standardized Regression Coefficient	Standard Error	Unstandardized Regression Coefficient	Standard Error	
	BETA	BETA	B	B	*p*
Free W			−907.794	291.4988	0.0143
Leg upswing (cm)	0.8274	0.1384	7.7734	1.3006	0.0003
Shin circumference (cm)	0.4158	0.1312	10.8388	3.4223	0.0132
Lower limb length (cm)	−0.4059	0.1409	−8.8995	3.0902	0.0205

**Table 9:** Summary details of the lambda ridge regression from the 2nd study stage for male participants; dependent variable = ranking points.

L = 0.10000, R = 0.95387189, R^2^ = 0.90987158					
F(6.4) = 0.7302 *p* < 0.04308, Estimated Standard Error: 32.695					
	Standardized Regression Coefficient	Standard Error	Unstandardized Regression Coefficient	Standard Error	
	BETA	BETA	B	B	*p*
Free W			−475.884	302.5304	0.1908
Leg upswing (cm)	0.3450	0.2251	3.3022	2.1549	0.2002
Complex reaction time: hands (s)	−0.3622	0.1589	−397.151	174.3357	0.0849
Bent arm hang (s)	−0.2708	0.1482	−2.0694	1.1325	0.1416
Bench press 1 RM test (kg)	−0.2183	0.1508	−1.4598	1.0085	0.2213
Hand punching combinations; uraken-uchi + gyaku-zuki, i.e., reverse fist + reverse punch (s)	0.3144	0.1990	2.5406	1.6078	0.1892
Simple reaction time (acoustic stimulation): hand (s)	0.2116	0.1924	585.9133	532.7161	0.3331

## Data Availability

The data presented in this study are available on request from the corresponding author. The data are not publicly available due to the consent provided by participants on the use of confidential data.
